# eggNOG 6.0: enabling comparative genomics across 12 535 organisms

**DOI:** 10.1093/nar/gkac1022

**Published:** 2022-11-18

**Authors:** Ana Hernández-Plaza, Damian Szklarczyk, Jorge Botas, Carlos P Cantalapiedra, Joaquín Giner-Lamia, Daniel R Mende, Rebecca Kirsch, Thomas Rattei, Ivica Letunic, Lars J Jensen, Peer Bork, Christian von Mering, Jaime Huerta-Cepas

**Affiliations:** Centro de Biotecnología y Genómica de Plantas, Universidad Politécnica de Madrid (UPM) - Instituto Nacional de Investigación y Tecnología Agraria y Alimentaria (INIA-CSIC), Campus de Montegancedo-UPM, 28223 Pozuelo de Alarcón, Madrid, Spain; Department of Molecular Life Sciences, University of Zurich, 8057 Zurich, Switzerland; SIB Swiss Institute of Bioinformatics, 1015 Lausanne, Switzerland; Centro de Biotecnología y Genómica de Plantas, Universidad Politécnica de Madrid (UPM) - Instituto Nacional de Investigación y Tecnología Agraria y Alimentaria (INIA-CSIC), Campus de Montegancedo-UPM, 28223 Pozuelo de Alarcón, Madrid, Spain; Centro de Biotecnología y Genómica de Plantas, Universidad Politécnica de Madrid (UPM) - Instituto Nacional de Investigación y Tecnología Agraria y Alimentaria (INIA-CSIC), Campus de Montegancedo-UPM, 28223 Pozuelo de Alarcón, Madrid, Spain; Centro de Biotecnología y Genómica de Plantas, Universidad Politécnica de Madrid (UPM) - Instituto Nacional de Investigación y Tecnología Agraria y Alimentaria (INIA-CSIC), Campus de Montegancedo-UPM, 28223 Pozuelo de Alarcón, Madrid, Spain; Departamento de Biotecnología-Biología Vegetal, Escuela Técnica Superior de Ingeniería Agronómica, Alimentaria y de Biosistemas, Universidad Politécnica de Madrid (UPM), Madrid 28040, Spain; Department of Medical Microbiology, Amsterdam University Medical Centers, Amsterdam, The Netherlands; Novo Nordisk Foundation Center for Protein Research, Faculty of Health and Medical Sciences, University of Copenhagen, 2200 Copenhagen N, Denmark; University of Vienna, Centre for Microbiology and Environmental Systems Science, Djerassiplatz 11030, Vienna, Austria; Biobyte solutions GmbH, Bothestr. 142, 69117 Heidelberg, Germany; Novo Nordisk Foundation Center for Protein Research, Faculty of Health and Medical Sciences, University of Copenhagen, 2200 Copenhagen N, Denmark; Structural and Computational Biology Unit, European Molecular Biology Laboratory, 69117 Heidelberg, Germany; Yonsei Frontier Lab (YFL), Yonsei University, 03722 Seoul, South Korea; Department of Bioinformatics, Biocenter, University of Würzburg, 97074 Würzburg, Germany; Department of Molecular Life Sciences, University of Zurich, 8057 Zurich, Switzerland; SIB Swiss Institute of Bioinformatics, 1015 Lausanne, Switzerland; Centro de Biotecnología y Genómica de Plantas, Universidad Politécnica de Madrid (UPM) - Instituto Nacional de Investigación y Tecnología Agraria y Alimentaria (INIA-CSIC), Campus de Montegancedo-UPM, 28223 Pozuelo de Alarcón, Madrid, Spain

## Abstract

The eggNOG (evolutionary gene genealogy Non-supervised Orthologous Groups) database is a bioinformatics resource providing orthology data and comprehensive functional information for organisms from all domains of life. Here, we present a major update of the database and website (version 6.0), which increases the number of covered organisms to 12 535 reference species, expands functional annotations, and implements new functionality. In total, eggNOG 6.0 provides a hierarchy of over 17M orthologous groups (OGs) computed at 1601 taxonomic levels, spanning 10 756 bacterial, 457 archaeal and 1322 eukaryotic organisms. OGs have been thoroughly annotated using recent knowledge from functional databases, including KEGG, Gene Ontology, UniProtKB, BiGG, CAZy, CARD, PFAM and SMART. eggNOG also offers phylogenetic trees for all OGs, maximising utility and versatility for end users while allowing researchers to investigate the evolutionary history of speciation and duplication events as well as the phylogenetic distribution of functional terms within each OG. Furthermore, the eggNOG 6.0 website contains new functionality to mine orthology and functional data with ease, including the possibility of generating phylogenetic profiles for multiple OGs across species or identifying single-copy OGs at custom taxonomic levels. eggNOG 6.0 is available at http://eggnog6.embl.de.

## INTRODUCTION

Comparative genomics has become a pivotal area of research in biology. By establishing the evolutionary relationships between genes from different species, we can use comparative analyses across genomes to transfer information between organisms, establish accurate phylogenies and identify clade- or species-specific traits.

Comparative genomics strives to achieve these goals by identifying homologous genes (those sharing a common ancestry) while attempting to further delineate speciation and duplication events that occurred during the evolutionary history of each gene family. Homologous genes that diverged after a speciation event are termed orthologs, while genes that originated after a duplication event are called paralogs. This evolutionary distinction between homolog subtypes has important practical implications and has been a matter of intense research during the past two decades (1,2). For instance, it is generally accepted that neo- and sub-functionalization events are most frequent between paralogs (i.e. after gene duplication) (3). By contrast, orthologs, and particularly one-to-one orthologs, tend to have conserved functions (4), even at large evolutionary distances (5). Therefore, accurate identification of orthology relationships is crucial for many steps in genomic workflows, such as *in-silico* functional annotation of unknown genes, protein–protein interaction prediction and species phylogeny reconstruction.

However, because duplication and speciation events can occur multiple times during the evolution of a gene family, establishing accurate orthology and paralogy relationships at scale is a complex procedure that requires intensive computations and careful interpretation of the results. Therefore, a variety of bioinformatic methods have been developed to address the problem of orthology prediction, each with its inherent strengths and weaknesses.

From a theoretical point of view, molecular phylogenies provide the best framework to investigate the intricate evolutionary relationships between genes from multiple species. Phylogenetic trees allow the identification of accurate, fine-grained orthology and paralogy relationships based on the tree topology, in which duplication and speciation events are associated to specific internal tree nodes. As a result, phylogenetic analysis has become a common approach to establish pairwise relationships between one or several specific genes across multiple organisms, allowing researchers to further differentiate between one-to-one, one-to-many, and many-to-many relationships (6). However, reconstructing accurate phylogenetic trees and identifying duplication and speciation events is highly challenging and computationally demanding, especially when hundreds of sequences are involved. Ensembl Compara (7), PhylomeDB (8), Panther (9) and other online resources use this approach to scale phylogenetic reconstruction to small and medium-sized sets of species.

Alternatively, clustering methods have been developed that allow orthologous groups (OGs) to be inferred at a given taxonomic level without having to disentangle all paralogy relationships below that level. While this is computationally more efficient, it cannot distinguish between orthologs and in-paralogs within each OG. This problem can be partially resolved by inferring OGs hierarchically at different taxonomic levels, as addressed in orthology databases such as OMA (10), OrthoDB (11) and Hieranoid (12).

eggNOG is a bioinformatics resource that aims to provide orthology data for organisms from all domains of life, together with comprehensive, precomputed functional, comparative, and evolutionary information. eggNOG employs both of the two aforementioned methodologies (clustering and phylogenetic analysis) and combines their predictions to infer orthology reports at different levels of detail. First, eggNOG provides precomputed OGs across thousands of taxonomic scopes, covering the three domains of life: Bacteria, Archaea, and Eukaryota. Second, each OG is further analysed using phylogenetic reconstruction which provides fine-grained resolution of duplication and speciation events within each OG. Moreover, eggNOG offers detailed information about functional and evolutionary aspects of each OG, integrating evolutionary data, conserved sequence domains, and functional terms into summarised reports for each OG in an effort to maximise its utility to the user.

Here, we describe eggNOG 6.0 database, which has been updated to include 12,535 reference species with up-to-date functional annotations from both existing and newly added sources. It also offers new online functionality that enables users to perform comparative genomic analyses with ease, such as phylogenetic profiling and OG filtering based on functional and gene duplication profiles. The updated eggNOG website contains new visualisation options that facilitate the exploration of taxonomic distributions and large annotated phylogenies associated with OGs. Overall, eggNOG continues to provide both a global repository of structured data available for use in large-scale analyses and an online resource for daily look ups and protein sequence classification. In the following, we describe the major changes made to the database and website as part of this upgrade.

## DATABASE UPDATES

### More than two-fold increase in the number of reference species covered

eggNOG 6.0 was built based on the proteomes of 12 535 reference species, including 1322 eukaryotes, 10 756 bacteria and 457 archaea. Prokaryotic proteomes were obtained from the reference species dataset available in proGenomes 2.1 (13) Eukaryotic proteomes were updated using Ensembl and UniProtKB reference proteomes. A complete list of species and proteomes included is available in the eggNOG website's download section. Compared to the previous eggNOG version this represents a 2.5-fold increase in the number of species covered, now spanning a total of 88 prokaryotic and 15 eukaryotic phyla. Most importantly, this update includes species representatives from 76 phyla, 103 orders and 32 classes which were not represented in previous versions, providing functional annotation and protein classification capability for new non-model species.

### *De novo* delineation of orthologous groups for 1601 taxonomic clades

eggNOG 6.0 continues to perform OG calculations using the species-aware clustering algorithm originally described in (14). This approach triangulates the best reciprocal hits between protein sequences and aggregates connected triades into clusters of orthologous groups (COGs). For consistency with other well established resources, and prior to *de novo* computation of OGs, we first expanded manually curated OGs available from the arCOGs (15), KOGs (16) and COGs (17) databases. For this, we mapped eggNOG proteomes against the reference alignment of each COG, KOG and arCOG, leading to larger versions of the same COGs, KOGs and arCOGs, but keeping their original OG names. Then, the remaining sequences lacking direct assignments to COGs, KOGs, and arCOGs were analysed *de novo* using the COG clustering algorithm in an unsupervised manner and the SIMAP Smith–Waterman reciprocal hits data (18), producing a large set of non-supervised orthologous groups (NOGs). In total, eggNOG 6.0 contains 17 032 907 OGs distributed across 1601 taxonomic clades: 4643 COGs, 12 332 arCOGs, 4852 KOGs and 17 011 080 NOGs. Additionally, 614 535 OGs were generated by merging OGs from the three basal taxonomic levels (Bacteria, Archaea and Eukaryota), thus representing orthology relationships at the LUCA (Last Universal Common Ancestor) level.

The broad taxonomic granularity available in eggNOG 6.0 (i.e. 1601 taxonomic clades obtained from the NCBI taxonomy tree) allows users to choose the most appropriate level for their analysis. For instance, orthologous and paralogous relations between proteins from the songbirds *Parus major* and *Serinus canaria* should be delineated at the *Passeriformes* (songbirds) taxonomic level, one of the new orders in eggNOG 6.0. Previously, users would have had to rely on the less resolved OGs at the *Aves* class level.

### New sources for functional annotation

eggNOG OGs were functionally annotated using multiple databases (Table 1), including the following sources: PFAM (19) and SMART (20) annotations to inform about the domain architecture and sequence motifs of each OG member; CARD (21) and CAZy (22) as specialised annotation sources focused on antimicrobial resistance genes and carbohydrate metabolism enzymes, respectively; KEGG (23) to provide different levels of annotations such as metabolic pathways, reactions, and modules; and PDB terms (24) to provide links to the three-dimensional conformations of proteins. We also obtained gene names and descriptions by linking eggNOG proteins to the RefSeq (25) and UniProtKB (26) databases which were also used to retrieve Gene Ontology (27) terms from its three main ontologies (biological process, molecular function, and cellular component). Finally, GO terms were condensed into GO slim terms to facilitate functional summaries and interpretation.

**Table 1.** Number of OGs annotated by the different source databases. OGs can be annotated by multiple sources. * New in eggNOG 6

Source database	Archaea	Bacteria	Eukaryota	All OGs
BiGG*	3 499	55 478	26 100	85 077 (0.50%)
CARD*	0	2 692	42	2 734 (0.02%)
CAZy	4 811	149 573	69 929	224 313 (1.32%)
GO Slim*	99 013	2 244 774	3 316 629	5 660 416 (33.23%)
GO	248 659	5 218 961	5 699 693	11 167 313 (65.56%)
KEGG	136 708	2 310 290	2 471 461	4 918 459 (28.88%)
KEGG enzyme*	67 028	1 090 786	745 655	1 903 469 (11.18%)
KEGG pathway	76 683	1 290 367	1 373 997	2 741 047 (16.09%)
KEGG module	33 573	484 270	230 624	748 467 (4.39%)
KEGG reaction*	52 340	814 505	418 838	1 285 683 (7.55%)
PDB*	5 765	56 295	93 943	156 003 (0.92%)
PFAM	256 787	5 979 209	5 845 596	12 081 592 (70.93%)
SMART	146 886	3 812 633	4 589 163	8 548 682 (50.19%)
All sources	307 970	7 107 439	6 971 165	14 386 574 (84.46%)
All OGs	381 650	8 257 748	8 393 509	17 032 907 (100.00%)

Despite comprehensive functional annotation, many bacterial and archaeal OGs (>1.6M) could not be annotated with any KEGG or GO term. To improve the functional annotation of these prokaryotic OGs, we inferred their putative functional roles by reconstructing their genomic neighbourhood and retrieving information about KEGG pathways that might be phylogenetically conserved and overrepresented among their neighbouring genes (28). Briefly, for each OG with at least four sequences, genomic context was extracted from a window of four contiguous genes (two upstream and two downstream). For each sequence, unique KEGG pathways assigned to neighbouring genes were retrieved and used to compute a conservation score for the whole OG, defined as the frequency at which a given KEGG pathway is observed in the genomic neighbours of all OG members. Only KEGG pathways with a score higher than 75% were considered for functional annotation of unknown OGs, allowing us to annotate additional 182 591 OGs.

### Improved website usability

The eggNOG resource (database and website) is intended both for large bioinformatic applications and as a tool for quick look-ups of functional assignments and protein classification. With this new version, we have extended our repository of downloadable data to include OG definitions, functional annotations, duplication profiles, and hierarchy in easily parsable formats (JSON or TSV files). These changes were made in response to numerous user requests demanding greater access to bulk data.

The backend and frontend of the eggNOG website have also been remodelled in order to improve user experience. The new website provides quick and advanced search options capabilities for all its 54M proteins and 17M OGs. In this new version, OG identifiers, specific protein names, gene symbols and various sources of sequence aliases (e.g. RefSeq and UniProt IDs as well as accessions) can be searched for using a minimalistic, autocompleting search bar. When a search term is found in multiple species (e.g. HUGO gene symbols), users can employ in-line filters consisting of partial or complete species names. For instance, typing ‘P53 sap’ into the main search panel will suggest the P53 protein in *Homo sapiens* as a first match. Hence, searches immediately yield not only the matching OGs at different taxonomic levels but also the precompiled tables of pairwise orthology relationships of the query protein with all other species. Additionally, users can perform advanced searches using general functional terms and taxa constraints, allowing the retrieval of a list of OGs containing specific sequence domains, KEGG pathways and/or specific taxa.

**Figure 1.**
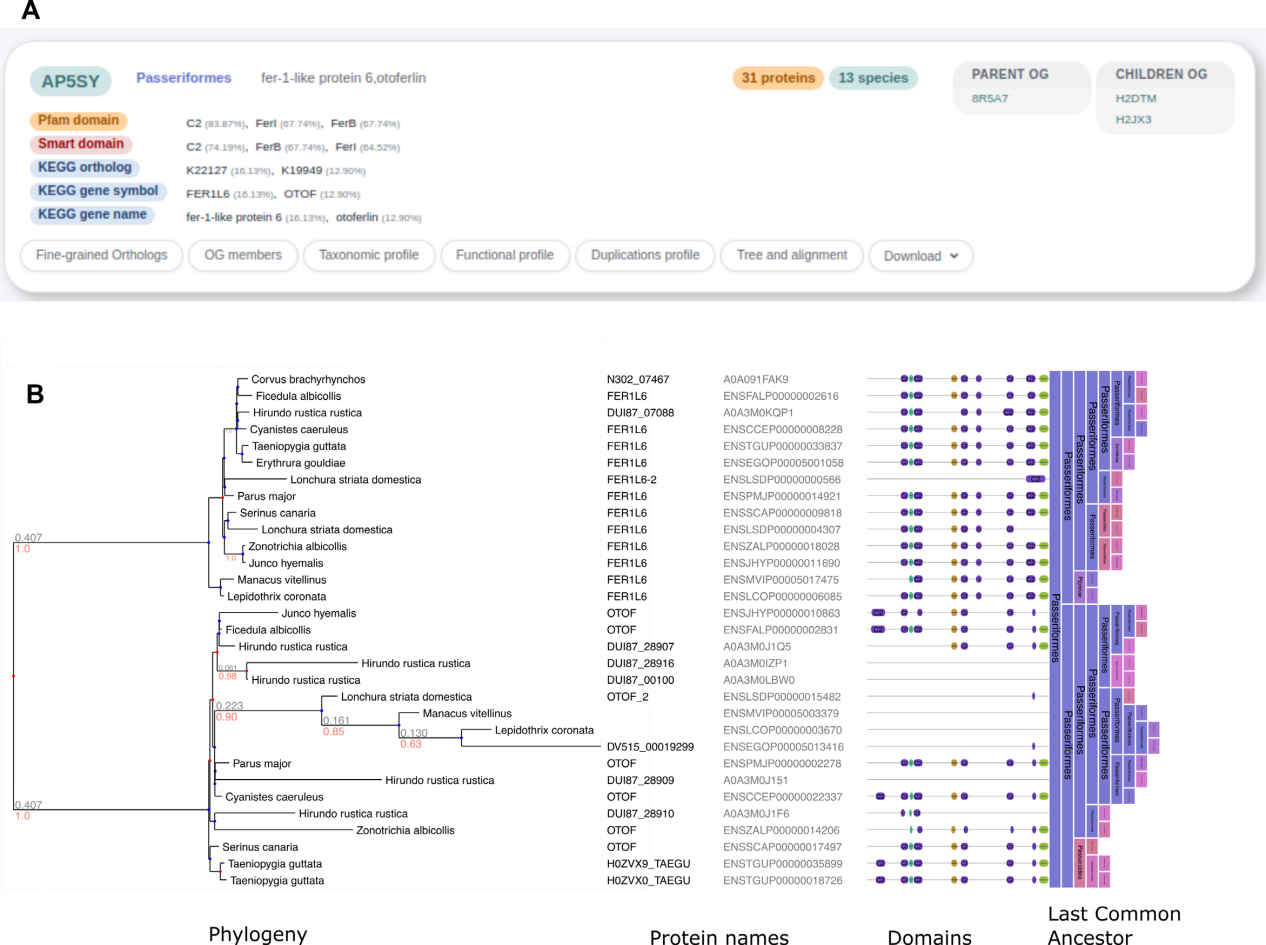
Summary card and extended information for the AP5SY OG. (**A**) OG general information, functional annotations summarised from various sources, and hierarchical information pointing to parent and child OGs. (**B**) Extended information about the OG using the new interactive visualisation tool for phylogenetic trees. Tree topology is annotated with duplication (red) and speciation (blue) events, branch length (grey), and bootstrap values (orange); common gene names are aligned with their corresponding branches, sequence domain structure is depicted schematically, while colored bands indicate the last common ancestor of each clade.

To facilitate a quick interpretation of results, the OGs matching a given query are now shown as a list of summary cards (Figure 1A) and the most important functional annotations are displayed and summarised in a more structured and informative way compared to previous versions. More detailed information about particular OGs will be revealed by clicking on the corresponding OG card buttons, including full details regarding OG members, taxonomic distribution, gene duplication events, OG hierarchy, and phylogenetic analysis. The same type of output can be obtained by querying individual protein sequences, which can be quickly classified into OGs using the sequence search panel.

Lastly, eggNOG 6.0 is continuously synchronised with other genomic resources such as STRING (29), eggNOG-mapper (30), proGenomes (13), SMART (20), GeCoViz (31) and others, with the aim to provide a federated network of bioinformatic tools where the underlying data, identifiers, and workflows are shared.

### Improved data visualisation

eggNOG 6.0 provides millions of phylogenetic trees, each disgraining the internal evolutionary relationships of proteins belonging to a certain OG. To cope with the increased size and complexity of these phylogenetic trees, the new eggNOG interface provides an interactive visualisation of fully annotated phylogenies using the newest (rolling) version of the ETE Toolkit (32) in combination with PhyloCloud (33) technology to handle the trees. This not only allows users to navigate the topology of large phylogenies but also enables eggNOG-specific visualisation layouts to display functional and evolutionary information across different tree branches (Figure 1B). At present, speciation and duplication events, PFAM/SMART domains, taxonomic classification, gene symbols and KEGG terms can be shown by enabling the corresponding layouts in the tree visualisation panel. Furthermore, the taxonomic distribution and taxa frequency within each OG is now provided using the interactive KRONA browser (34).

### Facilitating phylogenomic analyses

While the identification of single-copy orthologs or investigations of the presence/absence patterns of orthologs across multiple species are very common and highly useful analyses in comparative genomics, their high computational cost and complex bioinformatic workflows make them difficult to implement. eggNOG 6.0 alleviates this problem by providing a set of simple but powerful utilities that allow users to quickly identify OGs with specific patterns of gene duplication and generate phylogenetic profiles without the need of running custom bioinformatic pipelines.

**Figure 2.**
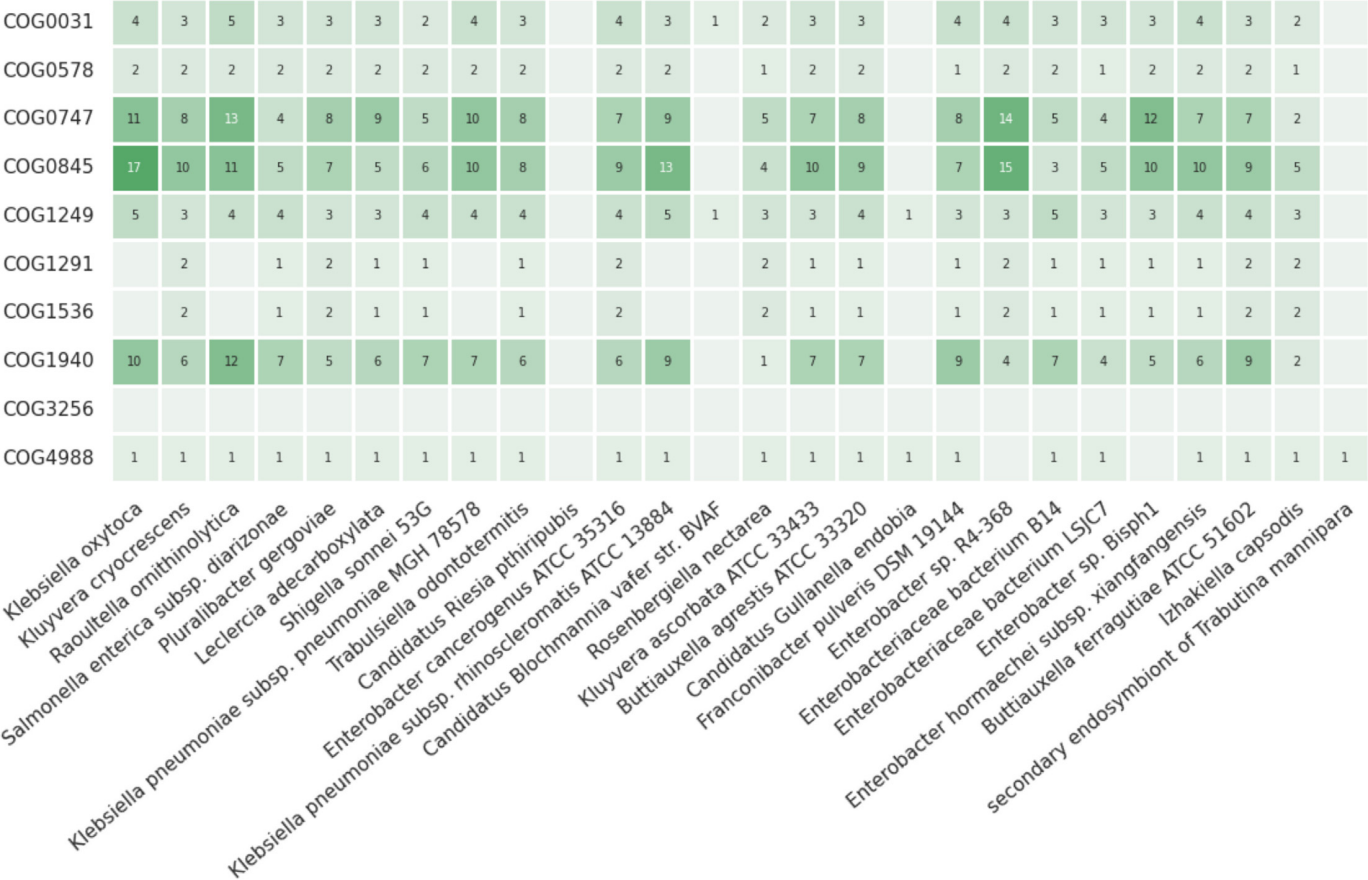
Distribution and copy number of genes belonging to 10 orthologous groups (COGs) associated with virulence factors across 26 Enterobacteriaceae species. The figure was generated automatically using the new eggNOG phylogenetic profiling functionality.

To produce a phylogenetic profile, eggNOG 6.0 only requires a list of OGs from the same taxonomic level and a set of target species of interest. A typical use case of this feature consists of the analysis of specific molecular functions (i.e. each represented by an OG) across multiple species (Figure 2).

### Conclusions and future perspectives

For over 15 years (35), eggNOG has been providing a consistent service to the scientific community by offering precomputed orthology assignments across up-to-date collections of fully-sequenced organisms. By combining orthology clustering with functional and phylogenetic analysis, eggNOG itself has also become a reference resource for phylogenomics studies, functional annotation of newly sequenced genomes, and protein classification, having served as the basis for thousands of genomic surveys to date.

With this new major upgrade to version 6, we expect eggNOG to be able to cope with an ever increasing number of genomes while providing improved representations of new taxonomic clades and facilitating future comparative genomic analyses. For the first time, eggNOG also offers *ad hoc* functionality to mine its data from a comparative genomics perspective, covering some of the most common requests from end users. However, given the high computational cost associated with each eggNOG release, and the exponential growth of newly discovered organisms (particularly from metagenomic studies), it may be necessary to implement a new set of algorithms and methods for orthology delineation in future releases.

## DATA AVAILABILITY

The data underlying this article are available for download at the eggNOG website, http://eggnog6.embl.de/.
